# Supplementary material for: Engineering of the CHAPk Staphylococcal Phage Endolysin to Enhance Antibacterial Activity against Stationary-Phase Cells
Source: Antibiotics (Basel). 2021 Jun 16;10(6):722. doi: 10.3390/antibiotics10060722 (PMC8235606; doi:10.3390/antibiotics10060722)
Supplement: Supplementary file 1 [file antibiotics-10-00722-s001.zip › antibiotics-1239774-supplementary-final.pdf]

antibiotics

MDPI

Supplementary Information

Engineering of the CHAPk Staphylococcal Phage Endolysin to Enhance Antibacterial Activity against Stationary-Phase Cells (Supplementary Information)

Sara Arroyo-Moreno [1], Máire Begley [1,2], Kornelia Dembicka [1] and Aidan Coffey [1,2,*]

[1] Department of Biological Sciences, Munster Technological University, T12 P928 Cork, Ireland; sara.arroyo-moreno@mycit.ie (S.A.-M.); maire.begley@cit.ie (M.B.); kornelia.dembicka@mycit.ie (K.D.)
[2] APC Microbiome Institute, University College, T12 YT20 Cork, Ireland
* Correspondence: aidan.coffey@cit.ie; Tel.: +353-214-335-486

```
ATG GCT AAG ACT CAA GCA GAA ATA AAT AAA CGT TTA GAT GCT TAT
 M   A   K   T   Q   A   E   I   N   K   R   L   D   A   Y
GCA AAA GGA ACA GTA GAT AGC CCT TAC AGA GTT AAA AAA GCT ACA
 A   K   G   T   V   D   S   P   Y   R   V   K   K   A   T
AGT TAT GAC CCA TCA TTT GGT GTA ATG GAA GCA GGA GCC ATT GAT
 S   Y   D   P   S   F   G   V   M   E   A   G   A   I   D
GCA GAT GGT TAC TAT CAC GCT CAG TGT CAA GAC CTT ATT ACA GAC
 A   D   G   Y   Y   H   A   Q   C   Q   D   L   I   T   D
TAT GTT TTA TGG TTA ACA GAT AAT AAA GTT AGA ACT TGG GGT AAT
 Y   V   L   W   L   T   D   N   K   V   R   T   W   G   N
GCT AAA GAC CAA ATT AAA CAG AGT TAT GGT ACT GGA TTT AAA ATA
 A   K   D   Q   I   K   Q   S   Y   G   T   G   F   K   I
CAT GAA AAT AAA CCT TCT ACT GTA CCT AAA AAA GGT TGG ATT GCG
 H   E   N   K   P   S   T   V   P   K   K   G   W   I   A
GTA TTT ACA TCC GGT AGT TAT GAA CAG TGG GGT CAC ATA GGT ATT
 V   F   T   S   G   S   Y   E   Q   W   G   H   I   G   I
GTA TAT GAT GGA GGT AAT ACT TCT ACA TTT ACT ATT TTA GAG CAA
 V   Y   D   G   G   N   T   S   T   F   T   I   L   E   Q
AAC TGG AAT GGT TAT GCT AAT AAA AAA CCT ACA AAA CGT GTA GAT
 N   W   N   G   Y   A   N   K   K   P   T   K   R   V   D
AAT TAT TAC GGA TTA ACT CAC TTC ATT GAA ATA CCT GTA AAA GCA
 N   Y   Y   G   L   T   H   F   I   E   I   P   V   K   A
ATG GTT AAT TCA TTT TCA AAT TCA ACT GCC CAA GAT CCA ATG CCT
 M   V   N   S   F   S   N   S   T   A   Q   D   P   M   P
TTC TTA AAG AGC GCA GGA TAT GGA AAA GCA GGT GGT ACA GTA ACT
 F   L   K   S   A   G   Y   G   K   A   G   G   T   V   T
CCA ACG CCG AAT ACA GGT TGG AAA ACA AAC AAA TAT GGC ACA CTA
 P   T   P   N   T   G   W   K   T   N   K   Y   G   T   L
TAT AAA TCA GAG TCA GCT AGC TTC ACA CCT AAT ACA GAT ATA ATA
 Y   K   S   E   S   A   S   F   T   P   N   T   D   I   I
ACA AGA ACG ACT GGT CCA TTT AGA AGC ATG CCG CAG TCA GGA GTC
 T   R   T   T   G   P   F   R   S   M   P   Q   S   G   V
TTA AAA GCA GGT CAA ACA ATT CAT TAT GAT GAA GTG ATG AAA CAA
 L   K   A   G   Q   T   I   H   Y   D   E   V   M   K   Q
GAC GGT CAT GTT TGG GTA GGT TAT ACA GGT AAC AGT GGC CAA CGT
 D   G   H   V   W   V   G   Y   T   G   N   S   G   Q   R
ATT TAC TTG CCT GTA AGA ACA TGG AAT AAA TCT ACT AAT ACT TTA
 I   Y   L   P   V   R   T   W   N   K   S   T   N   T   L
GGT GTT CTT TGG GGA ACT ATA AAG CTC GAG CAC CAC CAC CAC CAC
 G   V   L   W   G   T   I   K   L   E   H   H   H   H   H
CAC
 H
```

Figure S1. Sequence of the chimeric protein CHAPk-SH3blys. Red sequence corresponds to CHAPk, blue sequence corresponds to the SH3b cell-binding domain from lysostaphin, and purple sequence represents the His-tag region.

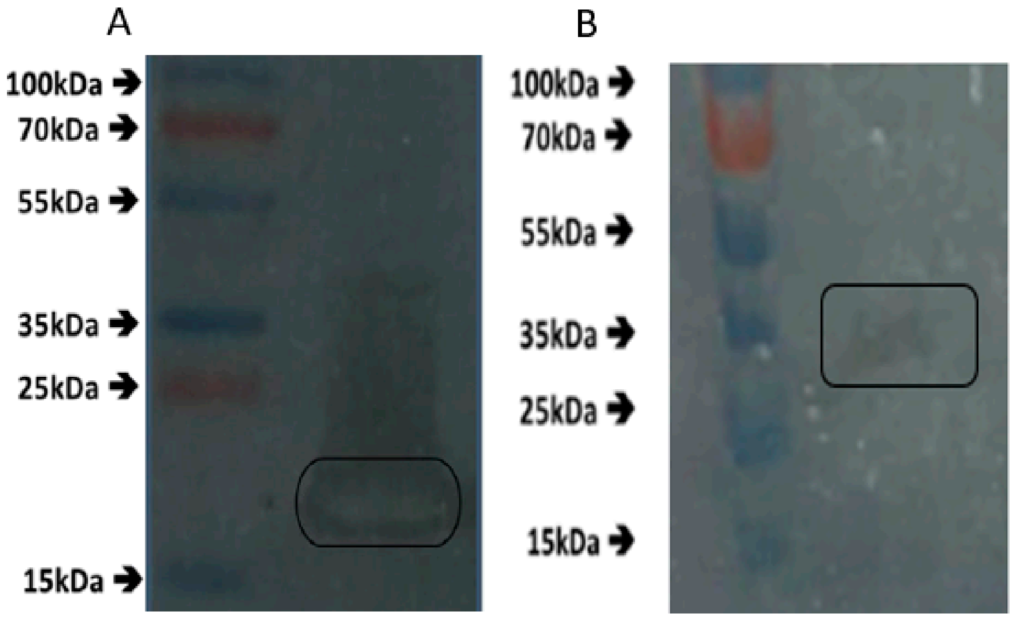

Figure S2. Zymogram using heat inactivated *S. aureus* DPC5246 cells, showing the lytic activity of; (A) CHAPk, having a band of clearing of approximately 20 KDa which corresponds to the predicted molecular mass of 18.6 kDa; (B) CHAPK-SH3blys, having a band of clearing of approximately 35 KDa which corresponds to the predicted molecular mass of 33.6 kDa.

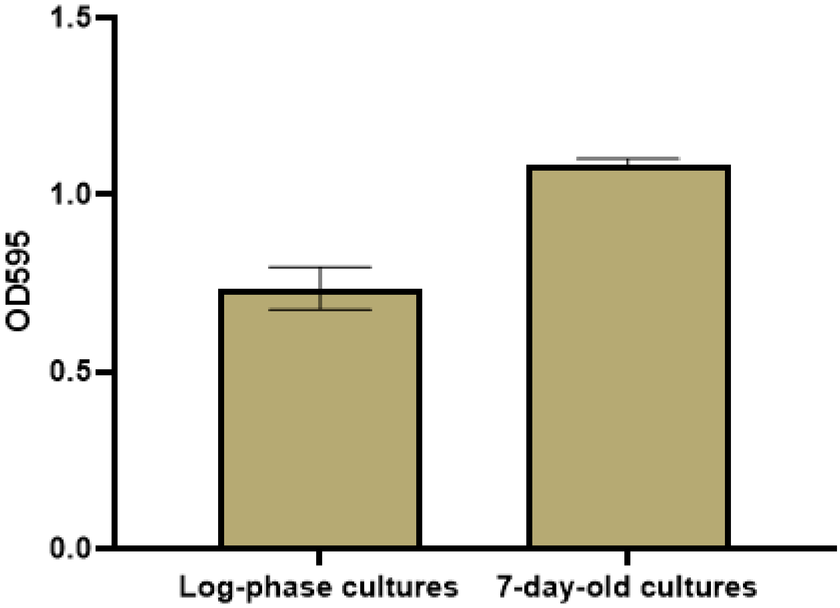

Figure S3. Density of biofilm cultivated from log-phase culture and 7-day-old culture. OD_{595} readings are the average of triplicates plus/minus their standard deviation. Biofilms were cultivated at 37ºC during 16h.

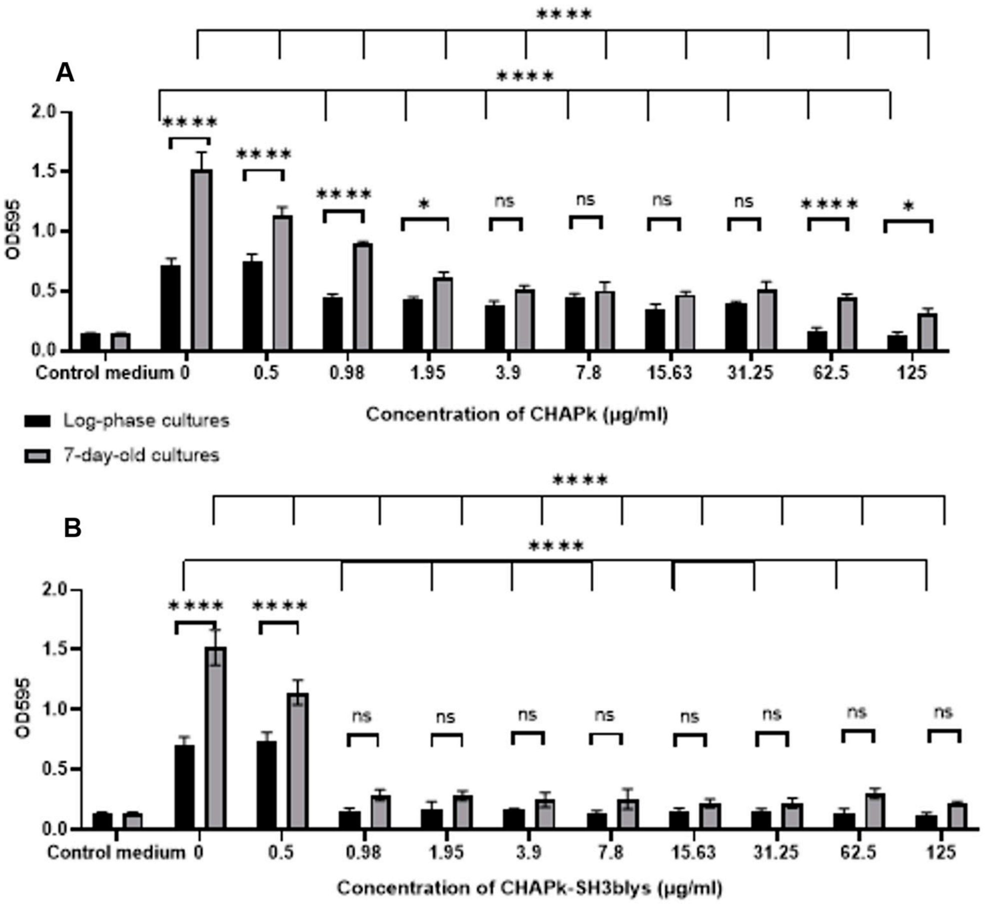

Figure S4. Biofilm prevention assay using concentrations of (A) CHAPk or (B) CHAPk-SH3blys ranging from 0.5 to 125µg/ml. OD_{595} readings are the average of triplicates plus/minus their standard deviation. p-values <0.0001 are represented by ****. No significant statistical differences are represented by ns. p-values comparing concentrations of CHAPk (between biofilms from log-phase and 7-day-old cultures) 1.95 and 125 represented by * were 0.022 and 0.0244, respectively.

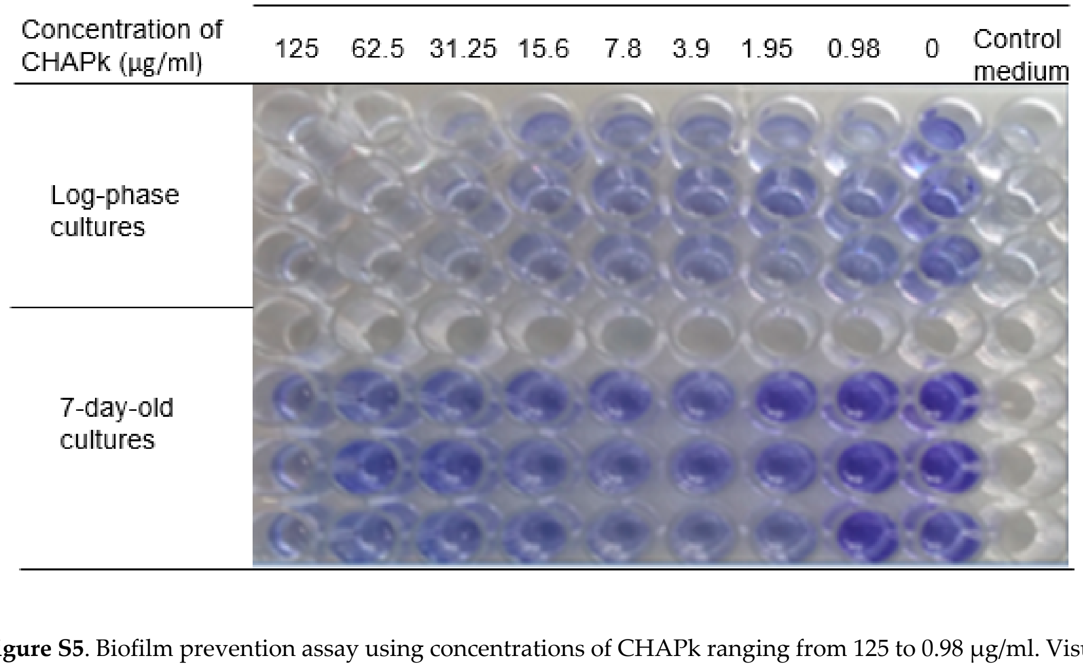

Figure S5. Biofilm prevention assay using concentrations of CHAPk ranging from 125 to 0.98 µg/ml. Visual representation of the 96-well plates after treatment with crystal violet.

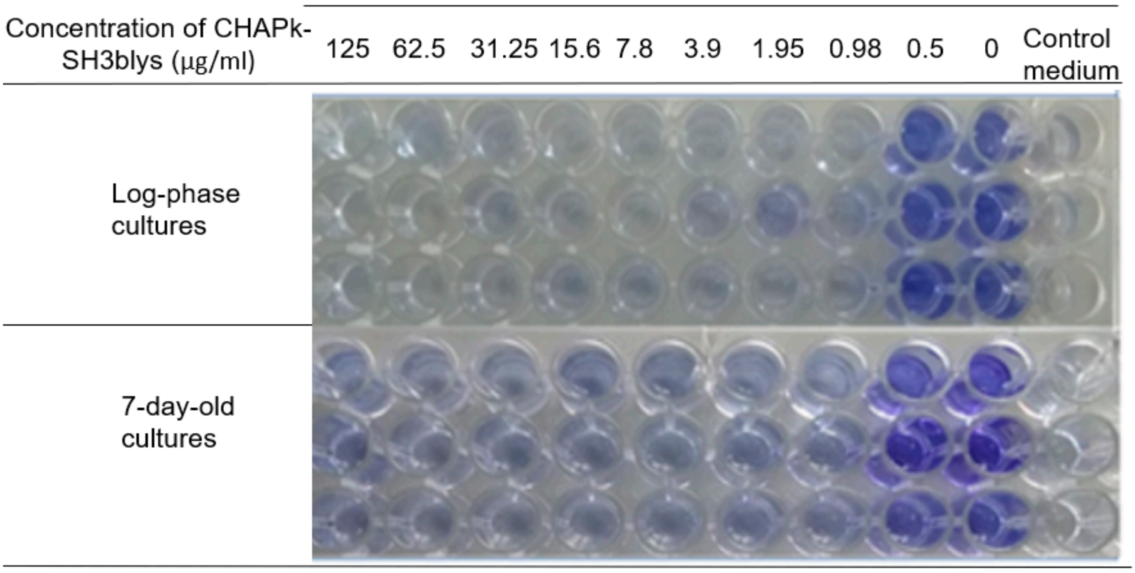

Figure S6. Biofilm prevention assay using concentrations of CHAPk-SH3blys ranging from 0.5 to 125 µg/ml. Visual representation of the 96-well plates after treatment with crystal violet.

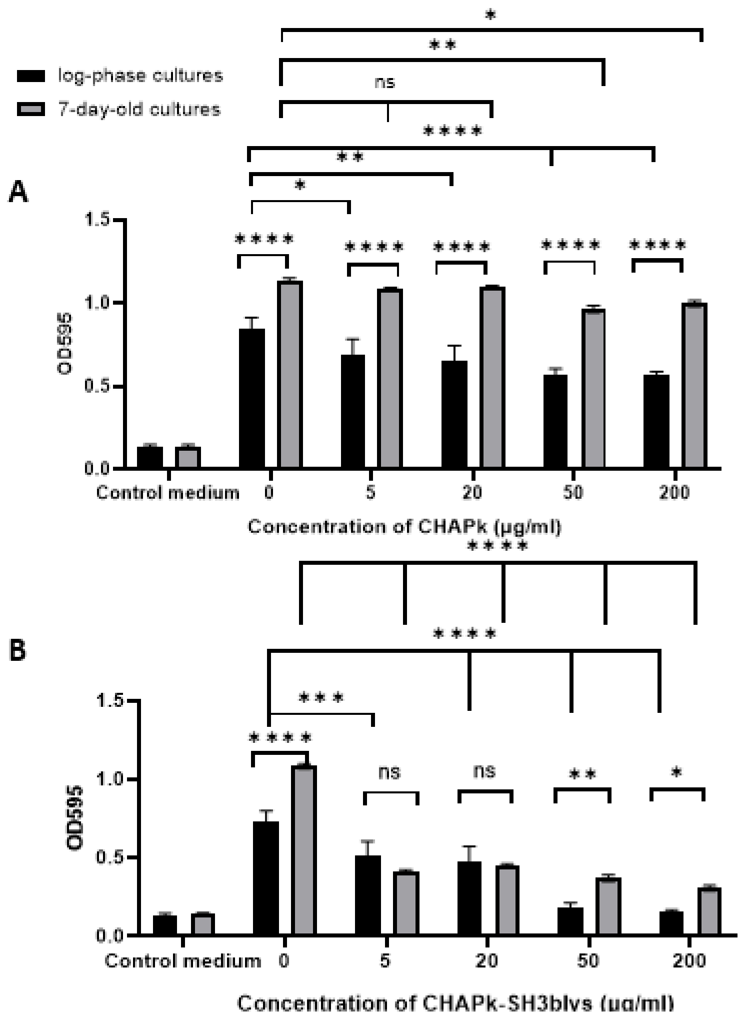

Figure S7. Biofilm disruption assay using *S. aureus* biofilms formed by cells in stationary and exponential phase. Concentrations of (A) CHAPk or (B) CHAPk-SH3blys, from 5 to 200 μg/ml, were used to disrupt biofilms. The disruption was determined by OD readings at 595nm. All readings are the average of triplicates plus/minus their standard deviation. P-values <0.0001 are represented by ****. No significant statistical differences are represented by ns. P-values comparing of untreated biofilm (formed from log-phase cultures) with concentrations of CHAPk of 5 and 20 μg/ml were 0.0136 and 0.011, respectively. P-values comparing untreated biofilm (formed from 7-day-old cultures) with concentrations of CHAPk of 50 and 200 μg/ml were 0.0060 and 0.043, respectively. P-value comparing untreated biofilm (formed from log-phase cultures) with a concentration of CHAPk-SH3blys of 5 μg/ml was 0.0002. P-values comparing CHAPk concentrations of 50 and 200 μg/ml (between biofilms from log-phase and 7-day-old cultures) were 0.006 and 0.043, respectively. P-values CHAPk-SH3blys concentrations of 50 and 200 were (between biofilms from log-phase and 7-day-old cultures) 0.0015 and 0.018, respectively.

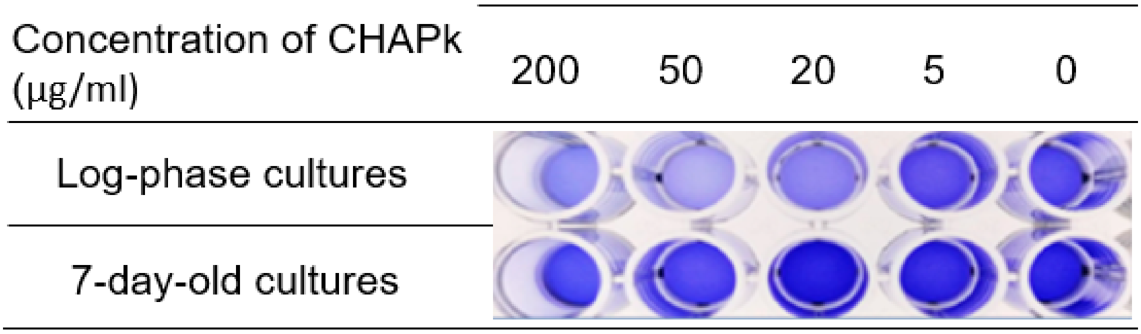

Figure S8. Biofilm disruption assay using *S. aureus* biofilms formed by cells in log-phase and 7-day-old cultures. Concentrations of CHAPk from 5 to 200 µg/ml, were used to disrupt biofilms. Visual representation of the 96-well plates after treatment with crystal violet.

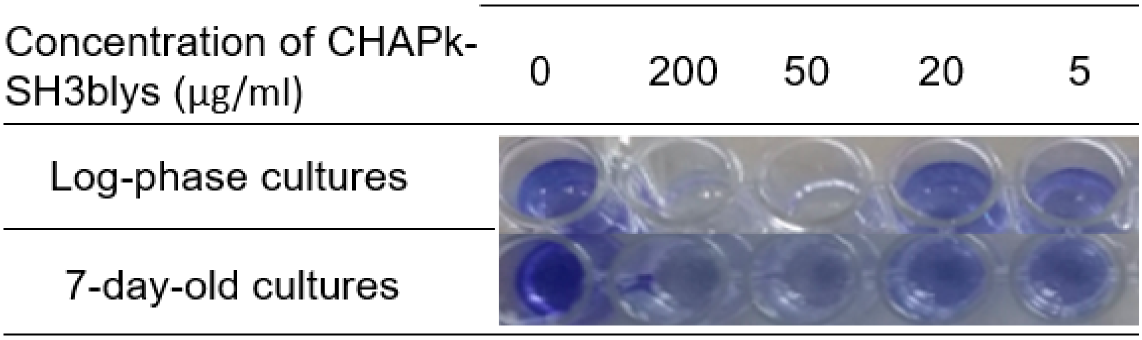

Figure S9. Biofilm disruption assay using *S. aureus* biofilms formed by cells in log-phase and 7-day-old cultures. Concentrations of CHAPk-SH3blys from 5 to 200 µg/ml, were used to disrupt biofilms. Visual representation of the 96-well plates after treatment with crystal violet.
